# Rootstock impacts on citrus flush dynamics, vegetative growth, and *Diaphorina citri* infestation and dispersion: Implications for huanglongbing management

**DOI:** 10.1002/ps.70929

**Published:** 2026-05-21

**Authors:** Deived Uilian de Carvalho, Eduardo Augusto Girardi, Wellington Ivo Eduardo, Haroldo Xavier Linhares Volpe, Fernando Alves de Azevedo, Marcelo Pedreira de Miranda, Renato Beozzo Bassanezi

**Affiliations:** ^1^ Fundo de Defesa da Citricultura (Fundecitrus) Araraquara Brazil; ^2^ Empresa Brasileira de Pesquisa Agropecuária (Embrapa) Mandioca & Fruticultura Cruz das Almas Brazil; ^3^ Instituto Agronômico de Campinas (IAC) Centro de Citricultura “Sylvio Moreira” Cordeirópolis Brazil

**Keywords:** Asian citrus psyllid, *Citrus* spp., HLB progress, horticultural practices, integrated pest management, vegetative growth

## Abstract

**BACKGROUND:**

Citrus tree vigor is regulated mainly by the rootstock. It may affect vegetative flushes and the dispersal and infestation of *Diaphorina citri* (Asian citrus psyllid) within the orchard, contributing to the inoculation of the huanglongbing (HLB) bacterium. We conducted an experiment using the sweet orange cv. Hamlin grafted onto two rootstocks of contrasting vigor: the vigorous ‘Sunki Tropical’ mandarin and the dwarfing ‘Flying Dragon’ trifoliate orange. The study aimed to investigate the impact of these rootstocks on tree growth, vegetative flush dynamics, psyllid infestation and dispersal, HLB progression, yield, and fruit quality in a young orchard.

**RESULTS:**

Psyllid infestation was strongly associated with the presence of flushes in a given period. Trees on vigorous rootstocks exhibited profuse growth, larger canopies, and longer, more abundant flushes, offering greater resources for psyllid feeding and reproduction over time. In contrast, trees on dwarfing rootstocks had smaller canopies and fewer, shorter shoots (50% shorter than vigorous), resulting in reduced psyllid abundance for these trees as a host in our study. HLB progression was slower in dwarfed trees throughout the evaluation period; however, by 43 months after planting (MAP), disease incidence and severity increased, resulting in similar levels between treatments. Our findings indicate that dwarfing rootstocks have the potential to integrate management strategies to reduce psyllid populations and, consequently, HLB progression under strict control of the insect vector, even in areas with high disease incidence.

**CONCLUSIONS:**

Dwarfing rootstocks, such as ‘Flying Dragon,’ significantly restrict vegetative growth and flush development, thereby reducing available feeding and oviposition sites for psyllids, offering a promising component in integrated HLB management strategies by lowering vector populations and disease spread potential. © 2026 The Author(s). *Pest Management Science* published by John Wiley & Sons Ltd on behalf of Society of Chemical Industry.

## Graphical abstract

Dwarfing citrus rootstocks restrict flush shoot growth on the scion, thereby reducing Asian citrus psyllid infestation, dispersal, and huanglongbing (HLB) progression. These findings support the use of dwarfing rootstocks as an effective cultural strategy in integrated HLB management for citrus orchards.

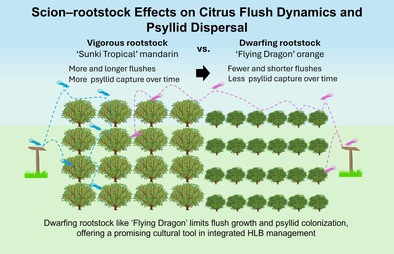

## 1 INTRODUCTION

Grafting has been used for centuries to propagate woody fruit trees.^1^ The effect of rootstock on tree vigor is one of the most commercially important examples of how the rootstock's genetics and intrinsic physiological traits can regulate scion behavior.^2^, ^3^ Rootstocks play a crucial role in citrus production, mainly because various horticultural traits, fruit quality, and resistance to biotic and abiotic stresses are remarkably influenced by grafting.^4^ Dwarfing rootstocks are frequently used to control tree size and enhance production efficiency across various fruit species.^5^, ^6^, ^7^ These rootstocks reduce the vegetative growth of the scion variety, resulting in trees that are easier to manage and harvest due to their reduced canopy volume.^8^

In this context, the ‘Flying Dragon’ trifoliate orange (*Poncirus trifoliata* var. *monstrosa* (T. Itô)) is a notable dwarfing rootstock variety that significantly reduces tree size in citrus.^9^, ^10^ This rootstock induces early bearing, enhances yield efficiency, and improves fruit quality in grafted trees.^11^, ^12^ It also facilitates more efficient harvesting, cultural practices, spraying operations, and high‐density plantings.^13^, ^14^ However, rootstock effectiveness can vary depending on the scion variety and growing conditions.^10^, ^13^, ^15^ In Brazil, ‘Flying Dragon’ is widely used as rootstock for ‘Tahiti’ acid lime (*Citrus* × *latifolia* (Yu. Tanaka) Tanaka), accounting for approximately one‐quarter of nursery trees produced in 2020–2022.^16^ However, its use in combination with sweet orange scions is still incipient. The rootstock ‘Sunki Tropical’ mandarin (*C. sunki* Hort. ex Tanaka), a chance seedling selection of ‘Sunki’ mandarin developed by Embrapa Cassava and Fruits' breeding program, promotes high vigor of the grafted scion, along with excellent yield and tolerance to citrus tristeza virus (CTV), blight, and drought.^17^, ^18^ Although it is graft‐compatible with most commercial scion varieties while producing high‐quality fruit,^19^, ^20^ ‘Sunki Tropical’ is mainly grafted with ‘Pera’ sweet orange in São Paulo State.^16^

The choice of rootstock strongly influences the vegetative vigor of the scion, which in turn can reduce the susceptibility of the trees to some pests and diseases.^21^, ^22^, ^23^ Nevertheless, this theme is seldomly studied, as in the case of the Asian citrus psyllid (*Diaphorina citri* Kuwayama; Hemiptera: Psyllidae) management, which transmits the bacterium ‘*Candidatus* Liberibacter asiaticus’ (*C*Las) associated with huanglongbing (HLB) disease.^24^ This insect vector is attracted to new flushes due to visual and olfactory cues.^25^, ^26^ Citrus flushes favor both the biology of the psyllid, influencing its population dynamics in the field, and the acquisition and transmission of *C*Las, which occur during its feeding behavior.^27^, ^28^, ^29^ This is of critical interest because citrus, particularly young trees, can produce multiple flushes throughout the year, driven by favorable environmental and management conditions.^30^ Given the influence of the rootstock on the citrus flushing pattern and growth,^31^ selecting scion–rootstock combinations with reduced vegetative vigor could reduce the HLB progress rate. A lower disease incidence was reported on trees grafted onto some dwarfing rootstocks in field conditions under strict psyllid control.^32^ However, the rootstock‐induced flush mechanisms related to vector preference and dispersal remain unknown.

To address these limitations, a field experiment was conducted with the early maturing ‘Hamlin’ sweet orange grafted onto vigorous (‘Sunki Tropical’ mandarin) and dwarfing (‘Flying Dragon’ trifoliate orange) rootstocks. This study aimed to investigate how scion–rootstock combinations with contrasting vigor affect the vegetative flush dynamics, Asian citrus psyllid infestation and dispersal, HLB progression, yield, and fruit quality within a young citrus orchard located in the Limeira region, southern São Paulo State, Brazil, where more than 70% of the trees of this region were affected by HLB during the experimental period.^33^ The implications of citrus grafting combinations for disease management and planting strategies are discussed.

## 2 MATERIALS AND METHODS

### 2.1 Plant material and experimental design

One‐year‐old nursery trees of the early‐season sweet orange (*Citrus × sinensis*) cv. Hamlin grafted onto vigorous (‘Sunki Tropical’ mandarin) and dwarfing (‘Flying Dragon’ trifoliate orange) nucellar rootstocks were planted in November 2021 at the experimental area. ‘Hamlin’ is a well‐known, vigorous, productive variety highly susceptible to HLB,^34^ while both rootstocks are HLB‐susceptible. Nursery trees were grown in 5.0‐L plastic bags in an insect‐proof screenhouse and pruned at 50 cm height before planting. Trees were planted at 6.0 m × 2.0 m (833 trees/ha) and 6.0 m × 1.5 m (1111 trees/ha) tree spacing for vigorous and dwarfing rootstocks, respectively. The experimental design was in randomized blocks with two treatments (vigorous and dwarfing rootstocks, respectively) and eight replicates, resulting in 16 plots. Within each block, plots were distributed with alternating treatments; that is, a dwarfing plot alternated with a vigorous plot, and so on. Each plot measured 50 m × 35 m and consisted of eight rows, with 17 and 23 trees per row for the vigorous and dwarfing treatments, totaling 136 and 184 trees per plot, respectively. The experimental area covered 2.63 ha with 2560 trees (see Supporting Information, Fig. S1 for details).

The experimental area was planted at the Centro de Citricultura ‘Sylvio Moreira’ of the Instituto Agronômico (IAC) located in Cordeirópolis (22°27′04″ S, 47°24′31″ W, and 718 m above sea level (a.s.l.)), in the Limeira region of the São Paulo State, Brazil. The on‐site climate of the region is classified as humid subtropical (Cwa) with dry winters and warm summers according to the Köppen–Geiger classification.^35^ Meteorological conditions were monitored daily for the entire experimental duration by an automatic meteorological station approximately 1.0 km away from the experimental area (see Fig. S2 for details). Trees were rainfed, and the water balance was calculated for the evaluated period according to the Thornthwaite and Mather^36^ method using an available water capacity of 100 mm. The soil in the experimental area is classified as Rhodic Hapludox with a clay texture.^37^

Nutritional management was based on the standard recommendation for sweet orange cultivation in the São Paulo State.^38^ Fertilization was based on the trees' needs, and nitrogen‐phosphorus‐potassium (NPK) fertilizers were applied three times a year during the rainy season (October–March). Weeds were managed with periodic mowing using an ecological mower between rows (≈ 4 times a year) and spraying systemic and residual herbicides in the rows, including glyphosate. Insecticide sprays to control psyllids were carried out fortnightly during the assessment period using insecticides present in the ProteCitrus list.^39^ Trees were not pruned during the evaluation period. The experimental area was situated amidst citrus orchards in a region with a progressively increasing incidence of HLB over the years: 61.8% in 2021, 70.7% in 2022, 73.9% in 2023, and 79.4% in 2024,^40^ reflecting an endemic disease condition.

### 2.2 Assessments

#### 2.2.1 Tree growth measurements

Vegetative growth measures were monitored approximately every 3 months from March 2023 to February 2024 (*n* = five assessments). A total of 256 trees (16 trees per plot, with four equidistant trees assessed from each of the four alternate rows per plot) were periodically evaluated (see Fig. S2 for details). Tree height and canopy diameter (parallel and perpendicular measures to the row) were measured with a graduated pole. The canopy volume was estimated according to Mendel.^41^ The growth rates (GRs) of tree height and canopy volume were calculated separately using the formula adapted from Carvalho *et al*.^42^:

$$\mathrm{GR}=\frac{\left|a_{2}-a_{1}\right|+\left|a_{3}-a_{2}\right|+\ldots +\left|a_{n}-a_{n-1}\right|}{n-1}$$

where *a*_1_, *a*_2_, …, *a*_*n*_, *a*_*n–1*_ = measurements of tree height (in meters) or canopy volume (in m^3^) at consecutively monthly intervals; and *n* = number of months.

#### 2.2.2 Vegetative flush dynamics and shoot length

The emerging new flushes on the tree canopy of both vigorous and dwarfing combinations were visually monitored fortnightly. The number of flushes per tree was counted, and each flush was classified according to a six‐stage phenological scale (V1–V6) as proposed by Cifuentes‐Arenas *et al*.^27^ A total of 256 trees (the same trees described in Section 2.2.1) were evaluated fortnightly from September 2022 to April 2024 (*n* = 36 assessments). The interval from V1 to V3 phenological stages was grouped to calculate the number and frequency (%) of new flushes over the assessment period, as psyllid shows reproductive preferences for young (V1–V3) over mature (V6) flushes.^27^ The flush frequency was calculated based on the relationship between the number of trees with new flushes (from V1 to V3) and the total number of evaluated trees in each assessment. The area under the number of the flush curve (AUNFC) was also calculated based on the number of flushes, which is given by the formula:

$$\text{AUNFC}=\sum_{i=1}^{n-1}\left(\frac{{\mathrm{NF}}_{i+1}+{\mathrm{NF}}_{i}}{2}\right)\times \left(T_{i+1}-T_{i}\right)$$

where NF_*i*_ corresponds to the number of flushes at stages V1–V3 in the *i*_th_ observation; and *T*_*i*+1_ *– T*_*i*_, is the number of days between observations.^43^ The area under the flush frequency curve (AUFFC) was also calculated similarly to the AUNFC.

The length of individual shoots was measured using a cloth measuring tape in two periods: March (fall) and October (spring) of 2023. A total of 16 shoots per plot were sampled (one shoot randomly picked per tree, the same trees described in Section 2.2.1), with measurements taken from the same trees evaluated for flush dynamics. Shoots were assessed at the V6 stage, characterized by fully expanded and mature growth.

#### 2.2.3 Infestation by *D. citri* adults (natural infestation)

The infestation of adult psyllids (*D. citri*) naturally present in the experimental area was monitored visually at fortnightly intervals from September 2022 to April 2024 (*n* = 36 assessments) using 30 cm × 10 cm double‐sized yellow sticky cards (YSCs; ISCA Technologies Ltd, Ijuí, Brazil). Cards were placed in the upper third of the tree canopy. These cards were positioned in trees from the periphery to the center of each plot, facing toward the orchard's edge. Within each row, cards were positioned at three distances from the plot edges (0, 18, and 36 m), with two replicate cards per distance (see Fig. S2 for details). Each plot consisted of evenly spaced arrays of six cards, resulting in 96 cards for each assessment. Cards were collected and replaced every 14 days. For each treatment, the mean number of adults per card (per tree) over 36 assessments corresponded to the *D. citri* infestation.

#### 2.2.4 Infestation and dispersal by marked and released *D. citri* adults

The marked *D. citri* release experiments were designed to evaluate whether adults exhibit differential preference and settlement between vigorous and dwarfing rootstock–scion combinations under field conditions. This approach aimed to determine how rootstock‐induced differences in tree vigor and canopy structure may influence psyllid distribution, with direct implications for HLB management strategies and grower decisions when establishing new orchards. Two release experiments were conducted, the first in early fall (23 March 2023) and the second in mid‐spring (24 October 2023), corresponding to 16 and 23 months after planting (MAP), respectively. *Diaphorina citri* adults (10–15 days after emergence) from a colony free of *C*Las were used for these experiments. This colony was maintained on *Murraya paniculata* (L.) Jack (Rutaceae) plants in a climate‐controlled room (temperature of 25 ± 2 °C; relative humidity (RH) of 60% ± 10%; and a 14 h:10 h light/dark photoperiod). Adults were marked with fluorescent powder (Day‐Glo Color Corp., Cleveland, OH, USA).^44^ Pink (Aurora Pink©) and blue (Horizon Blue©) fluorescent powder colors were used for each treatment (vigorous and dwarfing) in both release experiments (fall and spring). In each release period, one powder color was used to mark psyllids released in the vigorous treatment and the other for those released in the dwarfing treatment, according to the experimental design. Marked psyllids were confined on *M. paniculata* plants (*c*. 50 cm high) using mesh sleeve cages for a 72‐h acclimatization. After that, marked psyllids were released from a platform positioned 2.0 m above ground level, located 4.0 m from the center tree of the first row in each plot, resulting in 16 release platforms (see Fig. S1 for details). During each release event, 500 unsexed marked psyllids were released per plot in the morning (≈ 11:00 a.m.), resulting in a total of 16 000 marked psyllids across both experiments. Initially, *M. paniculata* branches with the marked psyllids were detached from the plants containing settled psyllids and attached to the top of each platform, and the cages were removed to release the insects. Weather conditions were monitored over time (see Fig. S3 for details) and during the release periods (see Fig. S4 for details).

After each release, visual inspections were performed on all trees to determine the number of marked psyllids per tree. All trees were assessed in each plot, totaling 2560 evaluated trees per assessment. Evaluations were performed 1, 3, and 7 days after release (DAR). Regardless of the color (pink or blue), marked psyllids were counted in all trees and plots to allow the identification of insects dispersing from a release platform into subsequent plots with a different rootstock treatment. The number of naturally occurring psyllids settled on trees was also recorded during the assessments. Insecticides were withheld 15–20 days before release to prevent psyllid mortality. Following the 7 DAR evaluation, chemical control was resumed. Heat maps were generated using psyllid counts recorded at each sampling location and interpolated across the experimental area to visualize adult dispersal and infestation intensity within and between plots of each evaluated rootstock at each assessment date.

#### 2.2.5 Huanglongbing (HLB) progress

Trees were monitored quarterly from February 2023 (15 MAP) to March 2024 (28 MAP) by visual inspections to identify typical HLB symptoms, including yellow shoots with mottled leaves, thickened leathery leaves with enlarged corky yellow veins, lopsided fruit, and premature fruit drop.^24^ Only experienced personnel from Fundecitrus scouted the trees. HLB incidence was determined based on visual identification of symptomatic trees at each inspection, and the cumulative HLB incidence was calculated as the sum of symptomatic trees recorded across all evaluation dates. The area under the disease progress curve (AUDPC) was also calculated similarly to AUNFC. The disease severity index (% of canopy area with symptoms) was assessed according to Bassanezi *et al*.^45^ at the end of the experiment in July 2024 (32 MAP) by scouting 16 HLB‐affected trees per plot (the same trees evaluated for tree growth). In addition, the incidence and severity of HLB‐affected trees were reassessed in June 2025 (43 MAP) on all trees in the experimental area to evaluate the long‐term disease impact on tree growth and productivity. In addition, leaves of the symptomatic Hamlin scion were sampled in April 2023 to confirm *C*Las detection levels of the evaluated graft combinations by quantitative polymerase chain reaction (qPCR) following analytical procedures described by Zheng *et al*.^46^ Sixteen trees of each graft combination were randomly selected across the experimental area and used as individual replications. A cycle threshold (Ct) < 34.0 was used to confirm the bacterial presence.

#### 2.2.6 Yield and fruit quality assessments

Fruits were harvested in May 2023 (18 MAP) and 2024 (30 MAP). During each harvest, the number of bearing trees per block was recorded, and fruits from all bearing trees were manually picked, pooled, and weighed using a digital scale (±0.01 kg). The percentage of bearing trees was used to estimate the yield per hectare (in kg/ha) for each treatment, providing a measure of reproductive performance. The yield efficiency was also determined based on the ratio between fruit yield (kg/tree) and canopy volume (m^3^/tree) assessed for each period for both cropping seasons. The results were expressed in kilograms per cubic meter (kg/m^3^) of the tree canopy.

For the 2023 cropping season, fruit and juice quality were evaluated. From each of the 16 plots (two treatments and eight blocks), 40 fruits were randomly collected from both HLB‐symptomatic and non‐symptomatic trees, resulting in one composite sample per plot for analysis. Then, harvested fruits were immediately transported to an industrial laboratory to undergo the physicochemical analyses. Fruits were weighed and then processed with an industrial juicer (391NSBR; John Bean Technologies Corp., Araraquara, Brazil) equipped with 3″ STD cups and standard components, following the manufacturer's specifications.^47^ After extraction, the fruit components, peel, core, pulp, and juice, were quantified. The proportion of each component was calculated based on its weight relative to the total fruit weight. With automatic temperature compensation, the total soluble solids (TSS) concentration was determined using a digital refractometer (RX‐5000α; Atago Co., Ltd, Tokyo, Japan). An aliquot of undiluted juice was used for this measurement, and results were reported in °Brix units. Titratable acidity (TA) was assessed by titrating the raw juice with a standard sodium hydroxide (NaOH) solution, using phenolphthalein as an endpoint indicator. TA values were expressed as grams of citric acid per 100 milliliters of juice (g/100 mL) following the Association of Official Analytical Chemists (AOAC)^48^ guidelines. The pH of the juice samples was measured with a digital pH meter (MS Tecnopon Special Equipment Ltd, Piracicaba, Brazil). Limonin in the juice was quantified using high‐performance liquid chromatography with diode array detection (HPLC‐DAD). The analysis was conducted on an Agilent 1260 Infinity LC System (Agilent Technologies, Santa Clara, CA, USA), following the procedures outlined by John Bean Technologies Corporation.^47^ Limonin concentration was expressed in micrograms per milliliter of juice (μg/mL). Additionally, the technological index (TI), representing the TSS content per standard orange box (weighing 40.8 kg), was calculated as described by Di Giorgi *et al*.^49^:

$$\mathrm{TI}=\frac{\mathrm{TSS}\times \mathrm{JC}\times 40.8}{10\;000}$$

where TI indicates technological index (kg TSS/box); TSS, total soluble solids (°Brix); JC, juice content (%).

The industrial yield (IY) was also calculated to estimate the number of orange boxes to produce one ton of frozen and concentrated orange juice (FCOJ) at 66 °Brix by the formula:

$$\mathrm{IY}=\frac{660}{\mathrm{TI}}$$

where IY indicates industrial yield (number of orange boxes to produce one ton of frozen and concentrated orange juice (FCOJ) at 66 °Brix); TI, technological index (kg TSS/box).

### 2.3 Statistical analysis and graphical visualization

Data from tree growth, flush dynamics, *D. citri* infestation, HLB progress and detection, yield, and fruit quality variables were submitted to the homogeneity of variance test using Bartlett's test and normality of residues using the Shapiro–Wilk's test at *P* ≤ 0.05. A one‐way analysis of variance (ANOVA) was then conducted to assess significant differences between the treatments (vigorous and dwarfing rootstocks) for each variable, using the *F*‐test to evaluate variations in group means. A two‐way ANOVA was also performed to evaluate the interaction between the treatment (vigorous *versus* dwarfing rootstocks) and assessed time (March *versus* October 2023) on shoot length at the V6 stage. The model also accounted for block effects by including block as a random factor. The number of evaluations that had the highest flush frequency and psyllids captured per card between the rootstock treatments was analyzed by the Chi‐squared (*χ* ^*2*^) test.

The number of marked and natural psyllids found in vigorous and dwarfing ‘Hamlin’ trees at different assessment dates after release was subjected to a repeated measures analysis using generalized linear mixed models (GLMMs) with quasi‐Poisson distribution. For the number of marked and natural psyllids, treatment and DAR were considered as fixed effects. In contrast, repeated measures in DAR and blocks were considered as a random effect, assuming an intercept that differed for individuals. Treatment and DAR effects were assessed by likelihood‐ratio tests between a complete model and a model without the effects of treatment or time. The same test was used to verify the interaction between treatment and DAR, comparing two models: one with interaction and one without. Generalized linear models (GLMs) with quasi‐Poisson distribution were used to analyze the effect of treatment on the number of marked psyllids found in the ‘Hamlin’ trees at each assessment date (DAR). The treatment effect on the number of marked psyllids on ‘Hamlin’ trees at each DAR was also analyzed. All analyses (*α* = 0.05) and graphical visualization were performed in R software version 4.4.0 (The R Foundation for Statistical Computing, Vienna, Austria) within the RStudio interface using the packages ‘ExpDes,’ ‘ggplot2,’ and ‘glmmTMB.’ Heat maps depicting the dispersal of marked and natural psyllids within the sweet orange orchard were created using Quantum Geographical Information System (QGIS) software version 3.38.3 (https://qgis.org/download/). Maps were correlated with the sample point by the square root inverse interpolation method of the distance between the points, considering the total number of adults per tree for each treatment (vigorous and dwarfing rootstocks), replicate (*n* = eight blocks), and time (March and October 2023) across all assessment dates (1, 3, and 7 DAR). Spearman's rank‐order correlation *ρ* (rho) was used to assess the relationship between distance from the release platform and the frequency of marked psyllids in each tree in the plot, as data from both vigorous and dwarfing treatments were not normally distributed across all evaluations (*P* ≤ 2.2 × 10^−16^, see Fig. S5 for details).

## 3 RESULTS

### 3.1 Tree growth measurements

**Figure 1:**
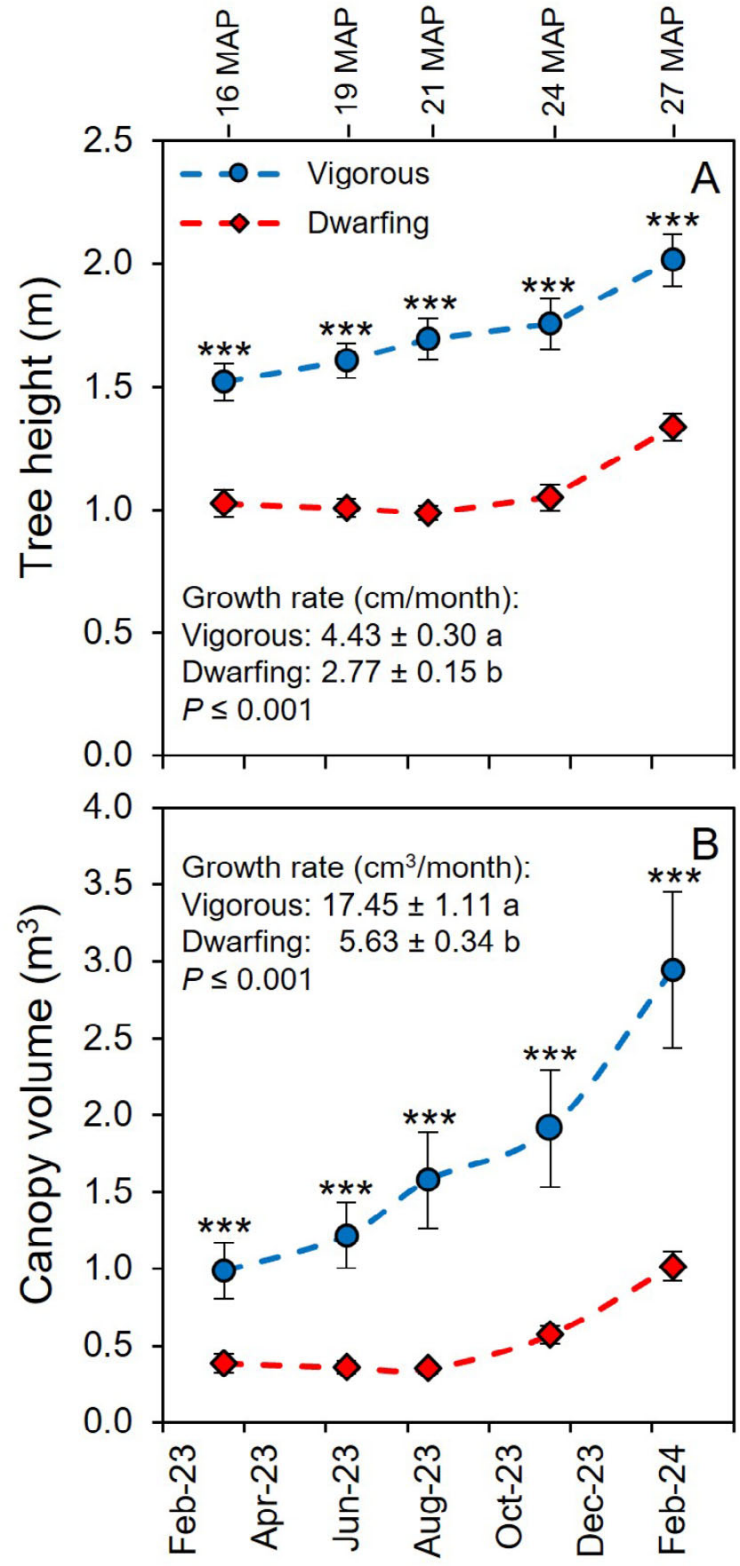
Means of tree height (A) and canopy volume (B) of young sweet orange (*Citrus × sinensis* cv. Hamlin) trees grafted onto vigorous (‘Sunki Tropical’) and dwarfing (‘Flying Dragon’) rootstocks on assessments from February 2023 (15 months after planting (MAP)) to February 2024 (27 MAP) in Cordeirópolis, São Paulo State, Brazil. Data are shown as mean and error bars (standard error of the mean). Asterisks (*) indicate the significance level of the differences between the tested treatments within evaluation dates and growth parameters according to the ANOVA (*F*‐test). Significance level: ***, *P* ≤ 0.001. Means followed by the same letter between rootstock treatments are not significantly different according to the ANOVA (*F*‐test) for the growth rates.

Significant differences (*P* ≤ 0.001) in vegetative growth were observed between the vigorous (‘Sunki Tropical’) and dwarfing (‘Flying Dragon’) rootstock treatments in all assessment dates for both tree height and canopy volume (Fig. 1). At the end of the evaluation (27 MAP, February 2024), trees from the vigorous rootstock were 1.5‐fold taller than those from the dwarfing. These differences were even more pronounced when calculating the canopy volume, with vigorous inducing a three‐fold larger canopy volume than the dwarfing rootstock. Over time, trees on vigorous rootstock exhibited higher growth rates for tree height and canopy volume compared to the dwarfing rootstock (Fig. 1).

### 3.2 Vegetative flush dynamics and shoot length

**Figure 2:**
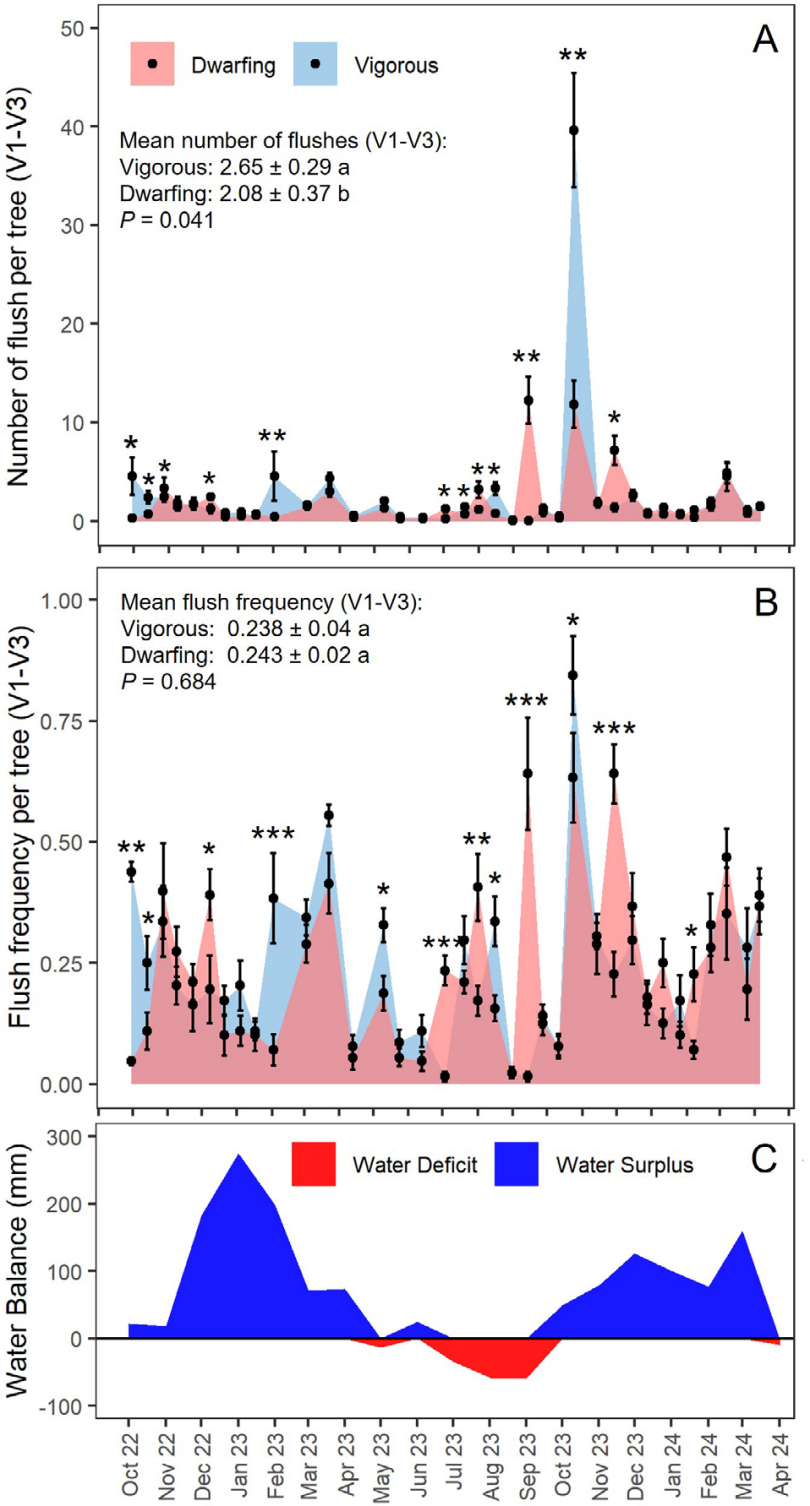
Mean number of flushes per tree (A) and flush frequency per tree (B) for V1–V3 stage flushes of young sweet orange (*Citrus × sinensis* cv. Hamlin) trees grafted on vigorous (‘Sunki Tropical’ mandarin) and dwarfing (‘Flying Dragon’ trifoliate) rootstocks from September 2022 to April 2024 in Cordeirópolis, São Paulo State, Brazil. Data are shown as mean and error bars (standard error of the mean). Asterisks (*) indicate the significance level of the differences between the tested treatments within evaluation dates according to the ANOVA (*F*‐test). Significance level: *, *P* ≤ 0.05; **, *P* ≤ 0.01; ***, *P* ≤ 0.001. Means followed by the same letter between treatments are not significantly different according to ANOVA (*F*‐test). Data that did not meet the assumptions of normality were transformed using the $\sqrt{x}$ method before submitting to ANOVA. (C) The water balance of the experimental area was calculated for the evaluation period.

The mean number of new flushes (V1–V3 flush stage) per tree varied throughout the assessment period (Fig. 2(A)). Over time, trees grafted on vigorous rootstock produced more flushes on average than those grafted on dwarfing one (*P* = 0.041), even though there was variation in the number of flushes per tree between assessment dates. This difference was also reflected in the area under the number of flush curve (AUNFC), which was greater for vigorous rootstock (1537) than for dwarfing rootstock (1148; P = 0.039). Despite these differences, no significant variation was observed in the average flush frequency between rootstocks (*P* = 0.684;Fig. 2(B)), corroborating the results obtained for the area under the flush frequency curve (AUFFC), for which both treatments showed similar values (133; P = 0.993), indicating similar numbers of trees with at least one new flush across treatments. From the total evaluations (*n* = 36), only 14 showed significant differences in the flush frequency between the treatments, in which 57% (*n* = 8) of the evaluations had a higher flush frequency in the vigorous treatment, while 43% (*n* = 6) scored higher for dwarfing (*χ* ^2^ = 1.47; df = 1; *P* = 0.225). The water balance was positive during most of the evaluation period, except from May to September, when it became negative, coinciding with the dry season (Fig. 2(C)).

**Figure 3:**
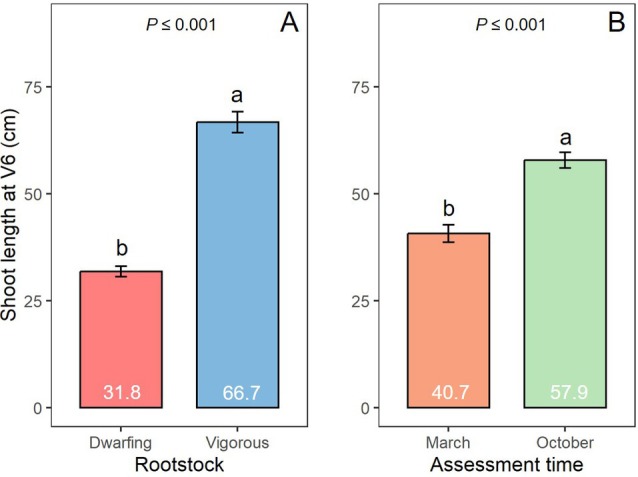
Mean shoot length at V6 stage flushes (cm) of sweet orange (*Citrus × sinensis* cv. Hamlin) young trees grafted on vigorous (‘Sunki Tropical’ mandarin) and dwarfing (‘Flying Dragon’ trifoliate) rootstocks during March (early fall, 16 months after planting (MAP)) (A) and October 2023 (spring, 23 MAP) (B) in Cordeirópolis, São Paulo State, Brazil. Data are shown as mean and error bars (standard error of the mean). Bars followed by the same letter within each rootstock and assessment time are not significantly different according to ANOVA (*F*‐test).

Regarding shoot length, there was no significant interaction between rootstock and assessment time (*P* = 0.057), indicating that the difference between vigorous and dwarfing rootstocks was similar at both assessment times (Fig. 3). However, significant main effects of both factors were detected. Overall, trees grafted on vigorous rootstock produced shoots that were twice as long, averaging 67 cm, compared to 32 cm for trees on dwarfing rootstock (*P* ≤ 0.001). Regardless of rootstock, shoot length also varied between assessment times, with shoots measured in October being 28% longer than those measured in March (*P* ≤ 0.001; Fig. 3).

### 3.3 Infestation by *D. citri* adults (natural infestation)

**Figure 4:**
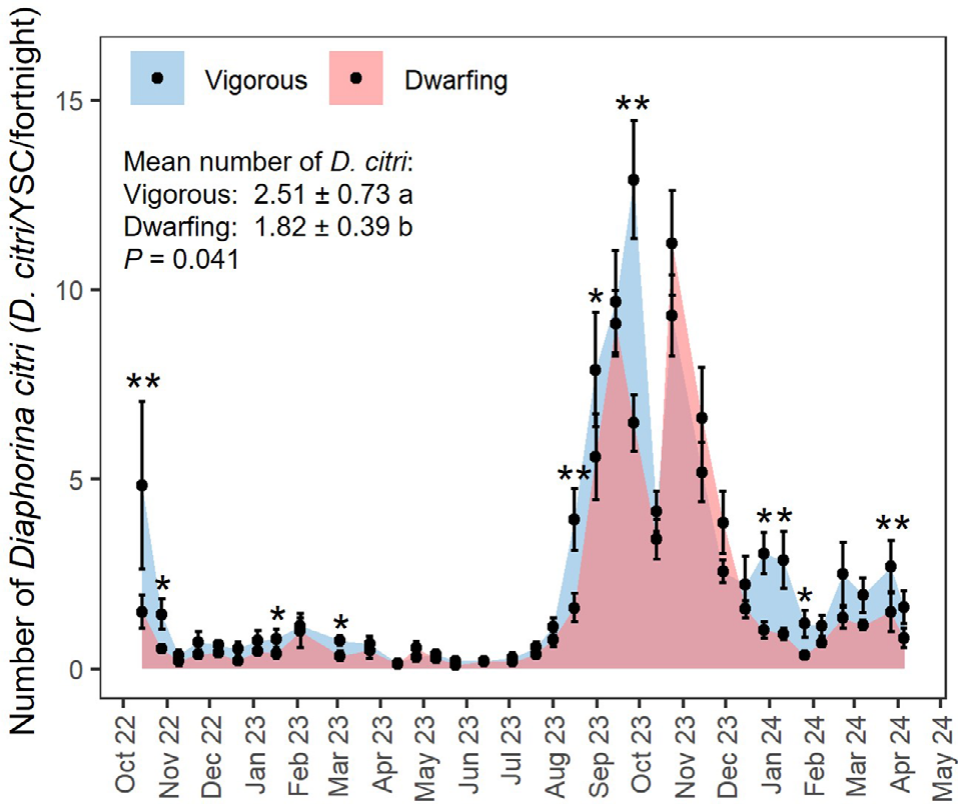
Mean (± standard error) number of *Diaphorina citri* adults/yellow stick card (YSC)/fortnight captured on young sweet orange (*Citrus × sinensis* cv. Hamlin) trees grafted onto vigorous (‘Sunki Tropical’ mandarin) and dwarfing (‘Flying Dragon’ trifoliate) rootstocks from September 2022 to April 2024 in Cordeirópolis, São Paulo State, Brazil. Data are shown as mean and error bars (standard error of the mean). Error bars followed by asterisks (*) or by different letters within treatments are significantly different according to the ANOVA (*F*‐test). Significance level: *, *P* ≤ 0.05; **, *P* ≤ 0.01. Data that did not meet the assumptions of normality and/or homogeneity of variances were transformed using the $\sqrt{x}$ method before submitting to ANOVA.

Across the entire evaluation period, the overall mean number of psyllids (*D. citri*) captures was significantly higher on trees grafted on vigorous rootstock (2.51) compared to those on dwarfing rootstock (1.82; *P* = 0.041), although differences between rootstocks were not consistently significant at all assessments, reflecting strong temporal variation in psyllid populations (Fig. 4). In 89% of the total assessments (*n* = 36), psyllid captures per card were higher in the vigorous rootstock treatment than in the dwarfing one (*χ* ^2^ = 40.5; df = 1; *P* ≤ 0.001), indicating that ‘Hamlin’ grafted onto ‘Sunki Tropical’ rootstock was in general more attractive to naturally occurring adults over the study period (Fig. 4). The highest capture rates occurred between August and November 2023, aligning with the natural population peak of psyllids in São Paulo State.

### 3.4 Infestation and dispersal by marked and released *D. citri* adults

**Figure 5:**
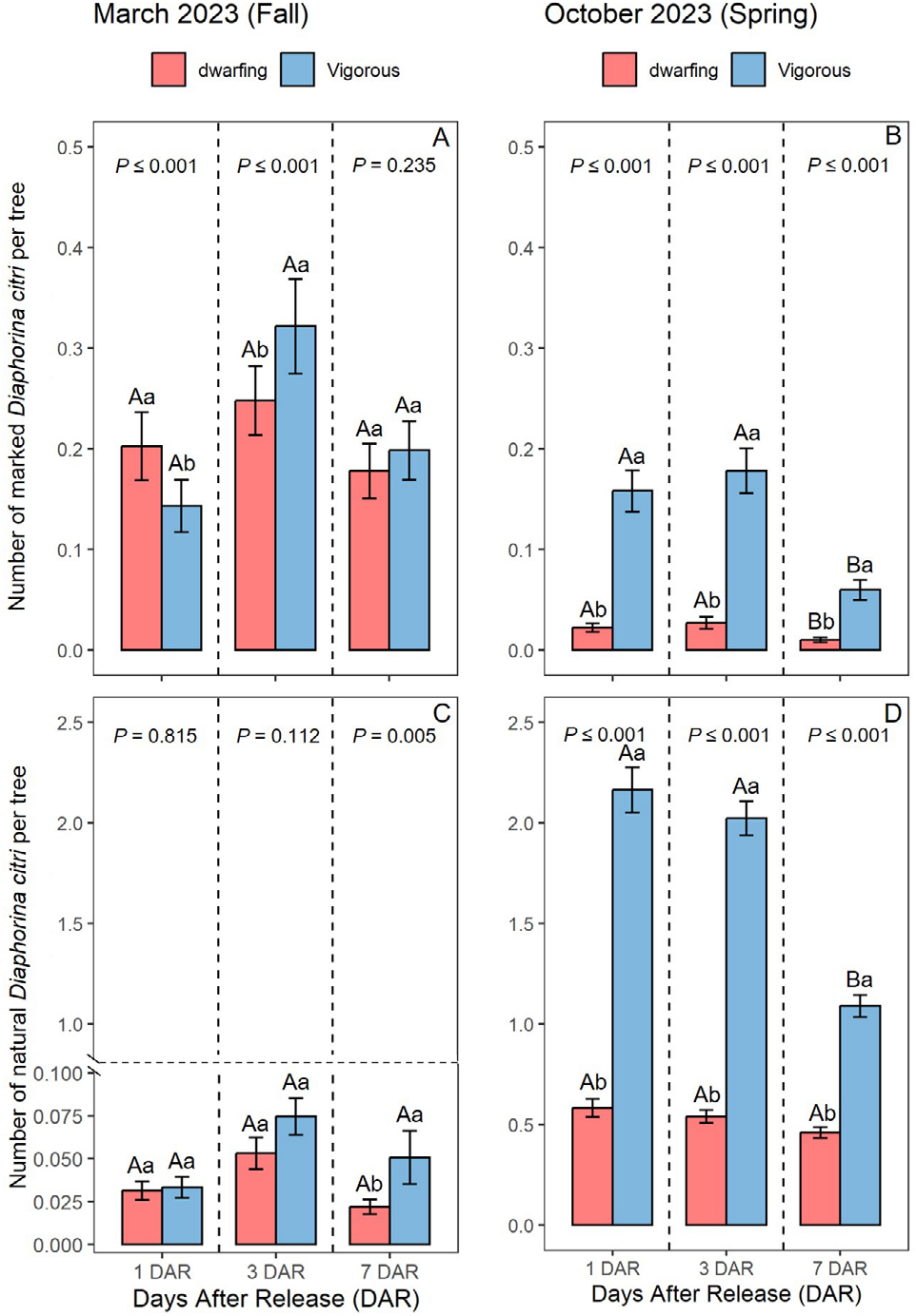
Mean number of marked (A and B) and natural (C and D) *Diaphorina citri* (Asian citrus psyllid) adults per young sweet orange (*Citrus × sinensis* cv. Hamlin) trees grafted onto vigorous (‘Sunki Tropical’) and dwarfing (‘Flying Dragon’) rootstocks in the evaluations performed 1, 3, and 7 days after release (DAR) of marked psyllids in the experiments conduct in March 2023 (fall; panels A and C) and October 2023 (spring; panels B and D) in Cordeirópolis, São Paulo State, Brazil. Data are shown as mean and error bars (standard error of the mean). Bars followed by the same lowercase and capital case letters between rootstocks within each DAR and between DAR within each rootstock, respectively, are not significantly different at *P* ≤ 0.05 according to ANOVA (*F*‐test).

In the March 2023 (early fall) experiment, the number of marked psyllids (*D. citri*) observed per tree was not influenced by the interaction between rootstock and DAR (*P* = 0.410; Fig. 5(A)). At 1 DAR, a higher number of marked psyllids per tree was observed in the trees with the dwarfed rootstock than those with the vigorous rootstock (*P* ≤ 0.001). In contrast, at 3 DAR, the number of marked psyllids per tree was greater on trees with the vigorous rootstock (*P* ≤ 0.001). By 7 DAR, no significant difference in marked psyllids per tree was observed between treatments (*P* = 0.235). While Fig. 5 presents the average number of marked psyllids per individual tree, the average number of marked psyllids per plot was also calculated to evaluate the infestation of the psyllid considering the tree population (see Supporting Information, Table S1 for details), and results are described here. At 1 DAR, a significantly higher average number of marked psyllids per plot was observed on dwarfing rootstock (37.3 ± 7.4) compared to vigorous rootstock (19.5 ± 4.9; *P* = 0.043). However, at 3 DAR, the total number of marked psyllids per plot was similar between dwarfing (45.6 ± 6.3) and vigorous treatments (43.8 ± 6.8; *P* = 0.686). Similarly, at 7 DAR, no significant difference was found between dwarfing (32.8 ± 4.1) and vigorous (27.0 ± 3.0; *P* = 0.251) treatments. Moreover, the number of migrating marked psyllids per plot, that is, those that moved from one plot to the opposite one (see Table S2 for details), was similar between treatments at 1 DAR (0.75 ± 0.3–1.75 ± 0.7; *P* = 0.154). However, at 3 DAR, the number of migrating marked psyllids was higher on vigorous (2.5 ± 0.5) compared to dwarfed plots (1.8 ± 0.3; *P* = 0.048), and this trend continued at 7 DAR, with more migrating marked psyllids in the plots with vigorous rootstocks (2.4 ± 0.8) than dwarfed ones (1.0 ± 0.4; *P* = 0.037).

In the experiment conducted in October 2023 (spring), the interaction between rootstock and DAR did not significantly (*P* = 0.632) affect the number of marked psyllids per tree (Fig. 5(B)). However, a five‐fold higher number of marked psyllids were found on vigorous trees compared to dwarfed ones at 1, 3, and 7 DAR (*P* ≤ 0.001). This trend was further supported by the total number of marked psyllids per plot (see Table S1 for details), in which the vigorous treatment had higher psyllid counts at 1 (21.5 ± 5.5; *P* = 0.013), 3 (24.3 ± 6.6; *P* = 0.015), and 7 DAR (15.8 ± 3.9; *P* = 0.012) compared to the dwarfing one (4.1 ± 1.3; 5.0 ± 1.9; and 2.5 ± 0.6 at 1, 3 and 7 DAR, respectively). Regarding the migrating marked psyllids, no differences were reported between treatments at 1 (≈2.8 ± 1.0; *P* = 0.660), 3 (≈0.5 ± 0.2; *P* = 0.289), and 7 DAR (≈0.7 ± 0.3; *P* = 0.095) (see Table S2 for details).

In addition to marked psyllids, the number of naturally occurring psyllids (unmarked) present in the experimental area was also monitored during the dispersal experiments (Fig. 5(C),(D)). In March 2023, the interaction between rootstock and DAR was not significant (*P* = 0.706) for the natural psyllids. The number of psyllids did not differ significantly between treatments at 1 (*P* = 0.815) and 3 DAR (*P* = 0.112) during this season. However, at 7 DAR, more natural psyllids were significantly found on vigorous trees than on dwarfed ones (*P* = 0.005; Fig. 5(C)). In October 2023, the interaction between rootstock and DAR was significant (*P* ≤ 0.001). The number of natural psyllids was consistently higher on vigorous trees compared to dwarfing at 1, 3, and 7 DAR (*P* ≤ 0.001; Fig. 5(D)). The presence of flushes per tree was also higher in this period of the year (Fig. 2(A)), notably for trees grafted onto the vigorous rootstock.

**Figure 6:**
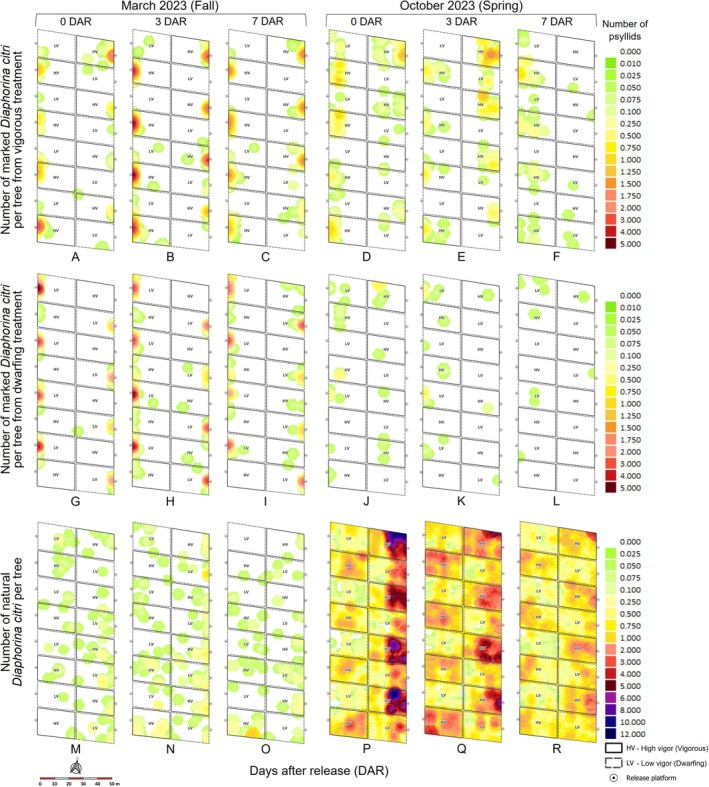
Heat maps illustrating the dispersal of marked (A–L) and natural (M–R) *Diaphorina citri* (Asian citrus psyllid) adults per young sweet orange (*Citrus × sinensis* cv. Hamlin) trees grafted on vigorous (‘Sunki Tropical’, panels A–F) and dwarfing (‘Flying Dragon’, panels G–L) rootstocks within the experimental orchard during evaluations performed 1, 3, and 7 days after release (DAR) of marked *D. citri* in the experiments conducted during March 2023 (fall) and October 2023 (spring) in Cordeirópolis, São Paulo State, Brazil.

To evaluate short‐range dispersal patterns relevant to local spread and edge effects, the relationship between distance from the release platform and the frequency of marked psyllids (*D. citri*) per tree was assessed in March 2023 (Fig. 6). A significant negative correlation was detected at 1 DAR for both vigorous (*ρ* = −0.324; *P* ≤ 0.001) and dwarfing (*ρ* = −0.368; *P* ≤ 0.001) rootstocks (see Fig. S5 for details), indicating that psyllid abundance declined with increasing distance from the release point. This trend became stronger at 3 DAR (vigorous: *ρ* = −0.411, *P* ≤ 0.001; dwarfing: *ρ* = −0.392, *P* ≤ 0.001) and remained significant at 7 DAR (vigorous: *ρ* = −0.394, *P* ≤ 0.001; dwarfing: *ρ* = −0.350, *P* ≤ 0.001), indicating a consistent decline in psyllid frequency with increasing distance, regardless of rootstock. In October 2023, however, the data did not display a consistent monotonic pattern across all evaluations, as shown by the irregular distribution of psyllids in the heat map (Fig. 6; see Fig. S5 for details). This uneven dispersal limited the effectiveness of Spearman's correlation in capturing the distance–frequency relationship during the spring (October) release experiment. Nonetheless, a general trend of decreasing psyllid frequency with distance was still noticeable, particularly in vigorous rootstock plots, in which the count of marked psyllids was clearly higher, while the pattern remained weaker and less consistent in dwarfing plots. Notably, the spatial distribution of naturally occurring psyllids was also irregular across the experimental area in both the fall and spring releases, even though more psyllids were found on vigorous rootstock plots (Fig. 6).

### 3.5 Huanglongbing (HLB) progress

**Figure 7:**
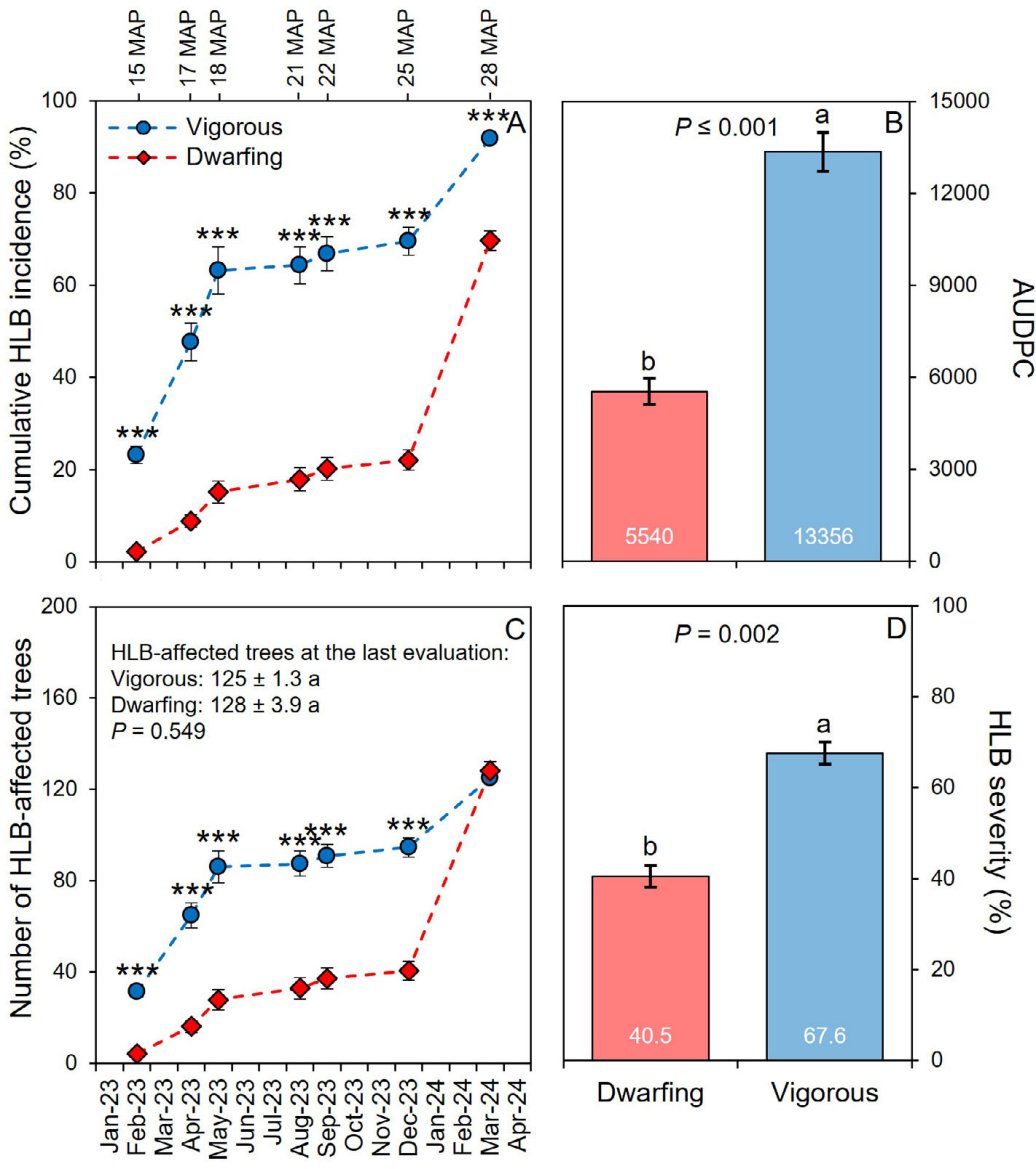
Cumulative huanglongbing (HLB) incidence (A), area under the disease progress curve (AUDPC; B), and number of HLB‐affected trees per plot (C) assessed for young sweet orange (*Citrus × sinensis* cv. Hamlin) trees grafted on vigorous (‘Sunki Tropical’) and dwarfing (‘Flying Dragon’) rootstocks from February 2023 (15 months after planting (MAP)) to March 2024 (28 MAP) in Cordeirópolis, São Paulo State, Brazil. (D) HLB severity was assessed in July 2024 (32 MAP) during the winter season. Data are shown as mean and error bars (standard error of the mean). Asterisk (*) indicates the significance level for treatment differences within evaluation dates (F‐test). Significance level: ***, *P* ≤ 0.001. Bars (panels B and D) and means (panel C) followed by the same letter are not significantly different at *P* ≤ 0.05 according to ANOVA (*F*‐test).

At the beginning of the evaluations, 15 MAP (February 2023), HLB incidence (expressed as the number of HLB‐symptomatic trees per total number of trees in a given area) was significantly lower (20‐fold) in trees grafted onto dwarfing rootstock compared to those on vigorous rootstock (*P* ≤ 0.001; Fig. 7(A)). By the end of the evaluation period (28 MAP), cumulative HLB incidence had increased four‐fold in vigorous trees and 32‐fold in dwarfing trees compared to the initial assessments. Despite this increase, HLB incidence remained significantly higher in trees grafted onto vigorous rootstocks throughout the study period, on average three‐fold higher during intermediate assessments (*P* ≤ 0.001). Consistent with these patterns, vigorous trees exhibited a greater disease burden over time, resulting in a significantly higher AUDPC compared with dwarfing trees (*P* ≤ 0.001; Fig. 7(B)). In contrast, the absolute number of HLB‐affected trees per plot (area) at the end of the evaluation period did not differ between treatments (*P* = 0.549; Fig. 7(C)). This apparent discrepancy reflects differences in planting density between treatments (833 trees/ha for vigorous rootstocks *versus* 1111 trees/ha for dwarfing rootstocks, a 33% higher density), such that similar numbers of symptomatic trees per plot corresponded to distinct incidence levels and disease progress on a per‐tree basis. In July 2024 (32 MAP), HLB severity was significantly higher in trees grafted onto the vigorous (67.6%) rootstock compared to those on the dwarfing rootstock (40.5%; *P* = 0.002; Fig. 7(D)), which may be related to the initial lower number of HLB‐symptomatic trees in the dwarfing treatment or differences in the moment of infection or symptoms appearance in each evaluated tree. However, 1 year later (43 MAP), HLB incidence and severity increased significantly across the experimental area, with nearly all trees showing symptoms. Vigorous and dwarfing combinations exhibited 90.4% and 82.9% HLB incidence, respectively (*P* = 0.008), and 90.2% and 86.3% severity, respectively (*P* = 0.053). A qPCR analysis was performed to confirm *C*Las detection in symptomatic scions in April 2023, with the Hamlin variety presenting similar Ct levels (18.12 ± 0.96) regardless of the rootstock used.

### 3.6 Yield and fruit quality assessments

**Figure 8:**
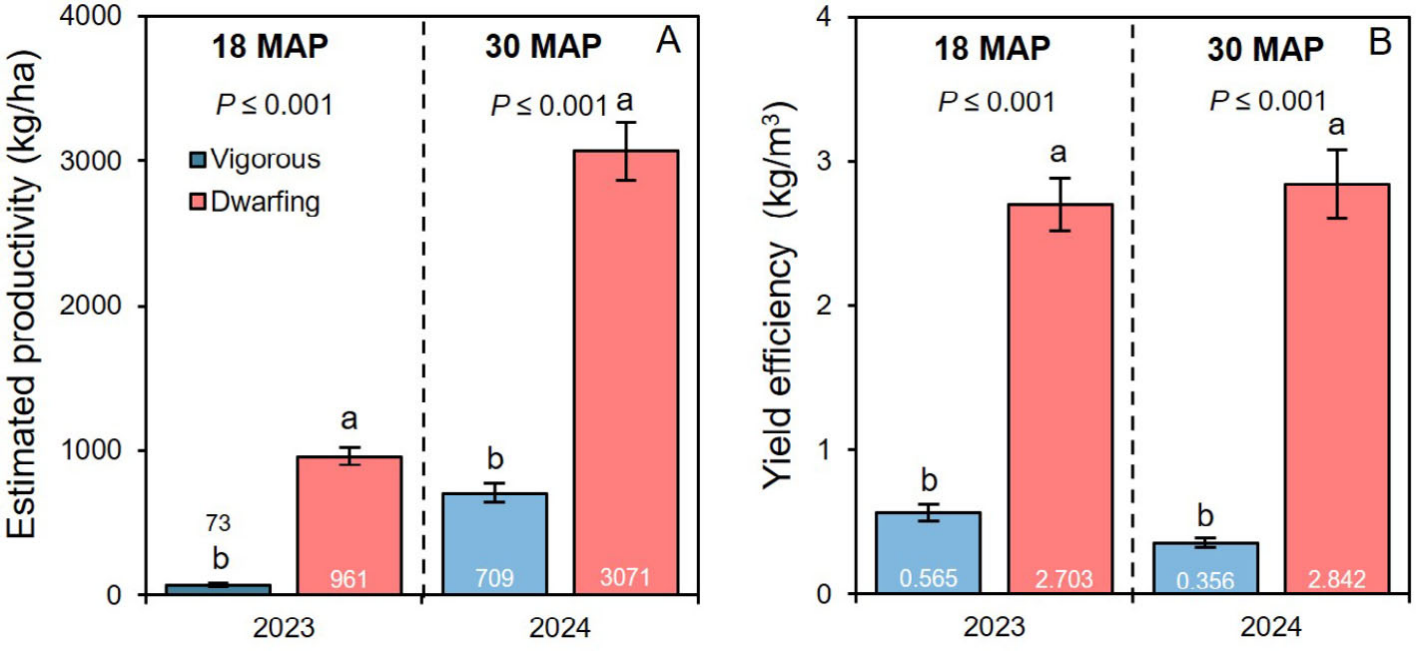
Mean estimated productivity (A) and yield efficiency (B) of young sweet orange (*Citrus × sinensis* cv. Hamlin) trees grafted onto vigorous (‘Sunki Tropical’) and dwarfing (‘Flying Dragon’) rootstocks 18 and 30 months after planting (MAP) in Cordeirópolis, São Paulo State, Brazil. Data are shown as mean and error bars (standard error of the mean). Bars followed by the same letter between rootstocks within the year (2023 and 2024) are not significantly different at *P* ≤ 0.05 according to ANOVA (*F*‐test).

At 18 MAP, the percentage of bearing trees was substantially higher (*P* ≤ 0.001) in trees grafted onto the dwarfing rootstock (86.6%) compared to vigorous (15.9%), leading to significantly higher estimated productivity (calculated on a per‐hectare basis using the percentage of bearing trees and the yield of individual trees) in the dwarfing treatment (*P* ≤ 0.001; Fig. 8(A)). This trend continued at 30 MAP, with 99.3% of dwarfing trees bearing fruit compared to 83.8% of vigorous trees, resulting in significant differences in estimated productivity between the treatments (*P* ≤ 0.001; Fig. 8(A)). Dwarfing trees demonstrated a 3.2‐fold increase in the estimated productivity from the first to the second year of cropping, while trees on vigorous rootstocks exhibited a 9.7‐fold increase over the same period. Additionally, dwarfing trees displayed significantly (*P* ≤ 0.001) higher yield efficiency (calculated as the fruit yield per unit canopy volume) at both 18 and 30 MAP compared to vigorous trees (Fig. 8(B)).

Fruit quality variables were assessed at harvest in June 2023, 18 MAP (Table 1). Fruits from dwarfing trees were 1.18‐fold heavier compared to those from vigorous trees (*P* ≤ 0.001). The proportion of different fruit parts varied significantly: fruits from vigorous trees had higher peel (*P* = 0.015) and core (seeds, membranes, and pomace) (*P* ≤ 0.001) contents compared to dwarfing ones. Conversely, the juice content was significantly higher in fruits from dwarfing than in those from vigorous trees (*P* ≤ 0.001). No differences were detected between treatments for the frit (peel fragments with essential oils) content (*P* = 0.261). Juice extracted from fruits of dwarfing trees had 1.22‐fold higher TSS content than vigorous (*P* ≤ 0.001). No variation was detected in TA between treatments (*P* = 0.944). The higher TSS content in dwarfing's fruit led to a higher TSS/TA ratio compared to vigorous rootstocks (*P* ≤ 0.001), as no differences were found in TA levels (Table 1). The pH level of juice was higher for dwarfing compared to vigorous treatment (*P* = 0.038). Similarly, dwarfing treatment had a TI 1.33‐fold greater than the vigorous one (*P* ≤ 0.001). Consequently, more boxes were required to produce one ton of FCOJ at 66 °Brix from vigorous compared to dwarfing rootstocks (*P* ≤ 0.001). Despite considerable variation within treatments for most quality parameters, limonin content in juice samples did not differ significantly between the evaluated rootstocks (*P* = 0.358).

**Table 1:** Mean values (± standard error of the mean) of quality variables of fruit harvested from young sweet orange (*Citrus × sinensis* cv. Hamlin) trees grafted onto vigorous (‘Sunki Tropical’) and dwarfing (‘Flying Dragon’) rootstocks in June 2023, 18 months after planting (MAP), in Cordeirópolis, São Paulo State, Brazil

Fruit quality variable	Vigorous	Dwarfing
Fruit weight (g)	159.3 ± 8.38 b	187.4 ± 8.96 a
Peel (%)	15.7 ± 0.77 a	13.6 ± 0.61 b
Core (%)	21.9 ± 1.00 a	17.9 ± 0.76 b
Frit (%)	6.21 ± 0.63 a	5.55 ± 0.77 a
Juice content (%)	55.8 ± 1.55 b	60.7 ± 1.52 a
Total soluble solids (TSS, °Brix)	6.98 ± 0.30 b	8.50 ± 0.26 a
Titratable acidity (TA, g/100 mL)	0.71 ± 0.03 a	0.71 ± 0.03 a
pH	3.54 ± 0.01 b	3.62 ± 0.06 a
Ratio (TSS/TA)	9.79 ± 0.52 b	11.90 ± 0.53 a
Technological index (TI, kg TSS/box)	1.59 ± 0.03 b	2.11 ± 0.03 a
Industrial yield (IY)^†^	415.8 ± 9.89 a	313.6 ± 5.84 b
Limonin (μg/mL)	2.47 ± 1.13 a	1.80 ± 0.65 a

*Note*: Means followed by the same letter in the row are not significantly different according to analysis of variance (ANOVA) *F*‐test.

^†^ IY, number of orange boxes to produce one ton of frozen and concentrated orange juice (FCOJ) at 66 °Brix.

## 4 DISCUSSION

This study confirms the importance of rootstock selection in regulating citrus tree vigor and its subsequent effects on *D. citri* (Asian citrus psyllids) population dynamics and HLB management. We demonstrated that rootstock vigor can influence tree growth patterns and psyllid population by comparing the vigorous ‘Sunki Tropical’ mandarin with the dwarfing ‘Flying Dragon’ trifoliate orange. The vigorous rootstock resulted in taller trees with larger canopies and more and longer flushes per tree, potentially offering greater feeding and reproduction sites for psyllids. In contrast, trees on dwarfing rootstocks were shorter, had smaller canopies, and produced fewer and shorter flushes over time, reducing the available area for psyllid activities. This resulted in lower psyllid population on ‘Hamlin’ sweet orange trees grafted onto dwarfing rootstock over time, suggesting its potential use as a planting strategy for improving HLB management (Fig. 4). Indeed, HLB progression in dwarfed trees was slower throughout the evaluation period compared with vigorous trees; however, by 43 MAP, HLB incidence and severity increased markedly across the experimental area, resulting in similar levels between treatments. This apparent convergence may partly reflect the relatively small plot size used in the experiment, which can increase edge effects and facilitate pathogen spread between treatments through vector movement. Under commercial orchard conditions with larger and more spatially continuous blocks, differences in HLB progression associated with canopy vigor and flush availability could potentially persist for longer periods.

Beyond structural differences, reduced vegetative vigor may also limit psyllid establishment in citrus orchards through physiological and biochemical pathways. Because new flushes constitute the primary sites for feeding and reproduction of psyllids, their abundance, duration, and nutritional composition directly regulate population growth.^50^ In fact, psyllids oviposit almost exclusively on young flushes (V1–V5), predominantly at the earliest stages (V1–V3), and when eggs are laid on more mature flushes (V4–V5), fewer than 10% successfully reach adulthood.^27^ Correspondingly, the risk of *C*Las transmission is closely linked to the flush stage, when infected adults feed on flushes at V2–V4, transmission efficiency is approximately 80%, whereas it drops to zero on fully mature shoots (V6).^28^ Therefore, it could be speculated that dwarfing rootstocks represent less biological potential for psyllid favorability because they induce a lower mean number and size of flushes. Probably, these reduced flushes tend to complete their development more rapidly, which could result in a short period that tissues remain suitable for oviposition and immature development, thereby limiting opportunities for psyllid population growth and *C*Las dissemination. Efficacy of insecticide spray coverage is also related to the growth rate of flushes and, consequently, psyllid control. Dwarfing rootstocks further influence this dynamic by altering carbon allocation patterns, increasing partitioning toward roots and reproductive sinks at the expense of vegetative growth.^51^ Such shifts in sink strength may reduce phloem flux to developing shoots (new flushes), potentially lowering concentrations of soluble sugars and free amino acids that are critical for phloem‐feeding insect development.^50^, ^52^, ^53^ Consequently, beyond simply reducing the number of flushes, dwarfing rootstocks may also decrease the nutritional quality of available feeding tissues. Rootstock‐mediated effects may further influence psyllid host location and acceptance through modifications in volatile organic compound (VOC) emissions. Flush development in citrus is associated with increased emission of terpenoids and other volatiles that mediate psyllid attraction.^54^ If dwarfing rootstocks reduce flush intensity or alter primary and secondary metabolism in developing shoots, the resulting changes in VOC profiles may be quantitatively or qualitatively modified, potentially decreasing host attractiveness to dispersing adults, as rootstock has been shown to influence leaf VOC composition in citrus.^55^ Hormonal signaling provides an additional integrative mechanism that connects growth regulation and defense expression. Rootstock–scion communication involving auxins and cytokinins functions as a regulatory driver of shoot initiation, elongation, and vegetative vigor through modulation of hormone synthesis and transport across the graft union.^56^, ^57^ At the same time, interactions between growth‐promoting hormones and jasmonates, key regulators of plant defense responses, govern growth–defense trade‐offs in many plant systems.^58^ Thus, rootstock‐dependent differences in hormonal balance may simultaneously regulate vegetative growth while enhancing constitutive or inducible defenses in developing flushes.

The reduced number of psyllids associated with dwarfing trees can be directly associated with the flush dynamics, which is crucial for the insect establishment on citrus.^27^ Our findings indicate that employing dwarfing rootstocks, which limit tree growth and the number and growth of flushes, can significantly reduce psyllid populations and HLB progress rate under an adequate psyllid management program. This effect was observed for 36 MAP, even under endemic disease conditions and in relatively small plots, in which more than 70% of HLB incidence was found in the region where the experiment was located.^40^ Therefore, implementing less vigorous scion–rootstock combinations in large orchards may be a complementary measure to reduce HLB incidence progress and improve orchard management practices in an area‐wide approach. Although psyllid dispersal across plots was similar regardless of rootstock variety and usually concentrated close to the release point, their infestation was higher on the vigorous combination on most assessment dates (Fig. 6). This may be associated with the abundance of flushes (Fig. 2). Therefore, relying solely on solid dwarf tree blocks may present challenges, as the limited flush availability of these trees could influence psyllid movement and preference, since infective adults tend to concentrate at the orchard edges after migrating from one to another citrus block under area‐wide management of HLB.^59^, ^60^ The lack of vigorous combinations could expose dwarf blocks to higher population pressures, emphasizing the need for a strategic deployment of scion–rootstock combinations. As an alternative approach to address this constraint, a mixed planting design could be considered using vigorous combinations along the orchard periphery and dwarfed trees in the center. This configuration may function as a ‘push‐and‐pull’ strategy for managing psyllid and other pests, as observed for other crops,^61^ and it should be further investigated in larger citrus blocks.

Dwarfing rootstocks can be integrated with additional cultural practices to optimize HLB management in modern citrus production. Low‐vigor citrus trees allow for higher planting densities,^8^ which can also reduce HLB's impact by altering disease spread dynamics.^62^, ^63^ High‐density planting increases the number of trees per unit area, potentially leading to a ‘dilution effect’ where disease incidence per area decreases as it dilutes among a higher number of trees.^62^ In our study, although the dwarfing treatment featured a 33% higher planting density compared to the vigorous rootstock, cumulative HLB incidence by December 2023 (25 MAP) was approximately three times lower. This evidences that HLB incidence is influenced not only by planting density but also by rootstock genotype, which may modulate key physiological and morphological traits. For example, the significantly reduced vegetative growth observed in trees grafted onto the true dwarfing ‘Flying Dragon’ rootstock, up to 65% less canopy volume compared to ‘Sunki Tropical,’ is consistent with previous findings^32^ and may facilitate and extend the use of individual protective covers (IPCs) in citrus orchards. These protective mesh bags effectively minimize the transmission of the HLB bacterium, particularly in new plantings or in areas where trees are replanted after removing infected and unproductive trees.^64^, ^65^, ^66^

Additionally, the use of dwarf trees has been associated with a reduced canopy volume per hectare,^8^ which may improve spray coverage during flushing periods and the economic feasibility of chemical control. Moreover, the canopy size and architecture of trees on dwarfing rootstocks may facilitate better penetration of spray droplets, increasing the likelihood of reaching internal targets, such as developing shoots and insect pests located within the canopy. Such improvement in spray deposition is particularly beneficial for managing pests and diseases that require high spray volumes and thorough coverage, such as citrus mite (*Brevipalpus yothersi* Baker).^67^ Therefore, beyond horticultural advantages, dwarfing rootstocks can play a strategic role in enhancing the efficacy of both chemical and biological control measures through better spray distribution. The application of processed kaolin^68^ and systemic insecticides^69^ may also be optimized by using a dwarf combination, which provides better coverage and insecticide distribution within the tree canopy, resulting in prolonged protection against psyllid.

Furthermore, in addition to their potential benefits for pest and disease management, young trees on the dwarfing ‘Flying Dragon’ rootstock also produced fruits of high quality. Fruits from dwarfing trees were heavier, with increased juice content, higher TSS, and a greater TSS/TA ratio, indicating enhanced flavor and sweetness,^10^ key attributes for juice processing and consumer preference.^70^, ^71^ The higher TI associated with dwarfing rootstocks further reinforces their suitability for industrial processing, as fewer fruit boxes are needed to obtain one ton of FCOJ,^49^ improving processing efficiency and reducing costs. In contrast, fruits from vigorous rootstocks had a higher proportion of peel and core during the early age of the trees, potentially reducing juice yield and impacting industrial quality. Overall, the improved juice quality and processing potential associated with dwarfing rootstocks emphasize their agronomic and industrial advantages for sweet orange production. However, the lack of significant differences in acidity and limonin content suggests that rootstock variety had minimal influence on fruit acidity and bitterness of ‘Hamlin’ sweet orange under the evaluated conditions. It is worth noting that fruit quality may have been influenced by the different incidence levels of HLB between rootstocks.

Despite the agronomic benefits associated with dwarfing rootstocks, their use in regions with endemic HLB and limited vector control, such as Florida (United States), poses specific challenges, as scions grafted onto small‐size‐controlling rootstocks may exhibit increased susceptibility to HLB compared to those on more vigorous rootstocks.^34^, ^72^, ^73^ This susceptibility raises concerns about the viability of dwarfing trees in areas with high infective psyllid populations, where disease control is particularly challenging. In fact, disease incidence and severity were very high for both rootstocks 1 year after experimental assessments ceased, corroborating this limitation. However, the compact size of these trees may streamline field operations for implementing citrus endotherapy using oxytetracycline (OTC)^74^ and other emerging molecules^75^ against HLB bacterium.

Another important consideration is that the dwarfing ‘Flying Dragon’ rootstock is more sensitive to drought than the vigorous ‘Sunki Tropical’ mandarin.^76^, ^77^ ‘Sunki Tropical’ employs more effective water conservation strategies and exhibits better genetic and physiological adaptations to drought, including osmotic adjustment and reduced oxidative stress.^65^ In contrast, ‘Flying Dragon’ experiences higher oxidative stress and less efficient water conservation^77^ despite its excellent yield and fruit quality performance under non‐irrigated conditions.^12^ This lack of drought tolerance may have restricted the flush shoot growth of dwarf trees during dry spells in our study (Fig. 2), likely influencing the photoassimilate translocation and the rate of leaf export.^78^ Such limitations may stem from rainfed cultivation practices, where trees generally initiate sprouting following rainfall after drought periods. Nonetheless, young trees typically show continuous flushing throughout the year,^30^ particularly in regions with well‐distributed rainfall, as observed in our study (see Fig. S3 for details), or in areas with supplemental irrigation. In this work, continuous flushing was observed for young trees grafted onto both rootstocks, which emphasizes the need for psyllid control during this orchard age. Since adult trees show more regular vegetative flushing, similar studies should also be performed comparing traditional and dwarfing rootstocks at mature age in combination with different scion varieties.

Furthermore, our results suggest that the reduced canopy volume induced by the dwarfing rootstock was not due to a lower flush frequency over time, but rather because they had fewer flushes per tree, resulting in shoots 50% shorter on average over the vigorous ‘Sunki Tropical’ rootstock (Fig. 3). Since yield efficiency was higher for trees on ‘Flying Dragon’ (Fig. 8), carbohydrate portioning between vegetative and reproductive structures may be related to the dwarfing phenotype as well. Consistent with our findings, Rodrigues *et al*.^32^ observed that different rootstocks did not significantly influence flush frequency in ‘Valencia’ orange trees; however, the abundance of flushes was lower when grafted onto ‘Flying Dragon’ and other semi‐dwarfing rootstocks. Notably, the flush dynamics of trees grafted onto the vigorous ‘Sunki Tropical’ rootstock may have been more adversely affected by the higher HLB incidence, which increased from 23% at the outset to 92% by 28 MAP. In contrast, dwarfing trees experienced an increase in HLB incidence from 2% to 70% up to 28 MAP, representing a sharper proportional increase over time despite a lower final incidence (Fig. 7(A)). Previous studies have confirmed that HLB‐affected trees tend to flush earlier^32^ and more frequently^79^ than healthy ones, which corroborates our findings. The differences in HLB incidence observed in our study align with the HLB severity data (Fig. 7(D)). While a higher severity in ‘Sunki Tropical’ pairings could suggest earlier bacterial infection, other factors may also explain the observed differences. These include the initial lower number of HLB‐symptomatic trees for ‘Flying Dragon’ treatment, delayed symptom expression (late infection), and/or reduced bacterial translocation due to potentially slower phloem flow in dwarfing pairings. However, severity increased substantially after 43 MAP, making the susceptibility to HLB of both graft combinations unequivocal. Bacterial detection in symptomatic trees was confirmed by qPCR and showed similar Ct values regardless of the rootstock, suggesting that rootstock genotype did not influence the response of the Hamlin scion. However, because infection occurred through natural transmission by infective psyllids, the inoculation date was not controlled during the evaluation period. Consequently, it was not possible to determine whether differences existed among rootstocks in terms of genetic tolerance or variations in disease latency and incubation periods, nor to assess the extent to which specific rootstocks might delay or mitigate the progression of HLB. Another limitation of this study was the need to suspend insecticide spraying during the release experiments of marked psyllids, which exposed trees to infection by natural psyllids and may explain the sharp disease detection in the late assessments.

Our findings indicate that dwarfing trees, with their reduced biological potential to generate new flushes, can decrease the available resources for psyllid feeding and reproduction and, consequently, *C*Las transmission under a stringent management scenario. In our study, psyllid infestation and dispersal were clearly associated with the abundance of flushes at a given period (Figs 2 and 6), which in turn were influenced by the rootstocks. Consequently, psyllid capture increased over time on the vigorous combinations, which exhibited more frequent and abundant flushes per tree (Fig. 3). This relationship confirms the relevance of vector control in different rootstock planting systems and, most importantly, corroborates the fact that flush dynamics is a fundamental aspect for the successful management of HLB and its vector, irrespective of varietal use. Future studies should explore the effectiveness of chemical controls on smaller flushes and evaluate how this factor can contribute to decreasing the HLB incidence progress in dwarfing rootstock orchards.

From a horticultural standpoint, our results align with previous studies indicating that ‘Flying Dragon’ trifoliate orange promotes early bearing and high fruit quality, even in the early stages of orchard development.^10^ The precocious nature of dwarfing trees enhances fruit set but restricts new flush development because of resource constraints caused by limited photoassimilates,^80^ a pattern consistent with our observations. Besides HLB management, dwarfing rootstocks are a critical component of modern citriculture. These rootstocks facilitate the establishment of high‐density orchards, enhance productivity, and improve operational efficiency in practices such as harvesting, pruning, and spraying.^8^, ^9^, ^14^, ^81^ In addition, dwarfing rootstocks show significant potential for mechanical harvesting.^82^ Importantly, the mechanisms underlying dwarfism in this rootstock variety appear to depend more on flush length and number than on flush frequency, indicating promising avenues for future research in citrus rootstock development.

In conclusion, adopting less vigorous scion–rootstock combinations, such as ‘Flying Dragon,’ offers a promising strategy for reducing HLB incidence progress and enhancing overall citrus orchard management practices. However, when dwarfing trees are planted in solid blocks, their reduced and more synchronized flushing, particularly at orchard edges where psyllid pressure is typically higher, may influence psyllid behavior and consequently disease spread, evidencing the need for strategic planting approaches that optimize scion–rootstock interactions and varietal distribution within orchards. Future research should investigate the long‐term effects of these practices on orchard health and HLB dynamics, while also refining integrated pest management strategies to ensure the sustainability of the citrus industry in the face of ongoing challenges. While our findings confirm the potential of ‘Flying Dragon’ trifoliate rootstock to reduce psyllid populations and HLB transmission under strict vector management, translating these results to commercial orchards requires careful consideration. Dwarfing trees can limit flush production, reduce canopy size, and improve spray coverage and operational efficiency, but these benefits may be offset by potential trade‐offs in yield under less intensive management or in environments with high psyllid pressure. Compatibility with current integrated pest management practices, including insecticide sprays, use of certified disease‐free scions, and area‐wide vector control, will be critical to ensure effectiveness. Regional adaptability is also a concern, as dwarf‐inducing rootstocks such as ‘Flying Dragon’ may be less tolerant to drought or other abiotic stresses in certain citrus‐growing regions, which could affect growth, productivity, pest, and disease incidence. Moreover, economic and horticultural barriers, such as higher initial costs for rootstock propagation, specialized planting designs, or irrigation requirements, may influence adoption by growers. Therefore, while dwarfing rootstocks offer promising advantages for the integrated HLB management and high‐quality fruit production, their real‐world implementation should consider site‐specific conditions, complementary management strategies, and economic feasibility to maximize translational impact.

## CONFLICT OF INTEREST

The authors declare that they have no known competing financial interests or personal relationships that could have appeared to influence the work reported in this article.

## Supporting information


**Figure S1.** Map of the experimental area (A), young sweet orange trees (*Citrus × sinensis* cv. Hamlin) grafted onto dwarfing (‘Flying Dragon’) and vigorous (‘Sunki Tropical’) rootstocks, photographed 16 months after planting (B), and release platform positioned 4 m from the first row of planting, oriented towards the center of the plot, used for the release of marked *Diaphorina citri* during experiments conducted in fall (March 2023) and spring (October 2023) in Cordeirópolis, São Paulo State, Brazil. (Source: Google Earth).
**Figure S2.** Map of the experimental area indicating the spatial distribution of sampled trees. For each plot, six trees were evaluated for *Diaphorina citri* capture using yellow stick cards (YSC) over time, and 16 trees were assessed for vegetative growth, flush shoot dynamics and length, and fruit yield. Tree sampling was conducted in both vigorous and dwarfing rootstock treatments according to the designated layout within each plot. Circles indicate the trees assessed for specific measurements: blue, trees assessed for *D. citri* capture by YSC; red, trees assessed for vegetative growth and flush growth. All plots (*n* = 16) were evaluated for these parameters according to the rootstock, although not all individual trees are circled in the figure for clarity.
**Figure S3.** Monthly average rainfall (mm) and air temperature (Max. Temp. = maximum temperature; Min. Temp. = minimum temperature; Mean Temp. = mean temperature) recorded at the experimental area located in Cordeirópolis, southern São Paulo State, Brazil. Data span from September 2022 to April 2024.
**Figure S4.** Daily rainfall and mean air temperature recorded during the release study of marked *Diaphorina citri* on young sweet orange trees (*Citrus × sinensis* cv. Hamlin) grafted onto vigorous (‘Sunki Tropical’) and dwarfing (‘Flying Dragon’) rootstocks. Data were collected during evaluations conducted 1, 3, and 7 days after release (DAR) in experiments held in the fall (March 2023) and spring (October 2023) in Cordeirópolis, São Paulo State, Brazil.
**Figure S5.** Relationship between the frequency of marked *Diaphorina citri* and distance (m) from the release platform on young sweet orange (*Citrus × sinensis* cv. Hamlin) trees grafted onto vigorous (‘Sunki Tropical’) and dwarfing (‘Flying Dragon’) rootstocks at 1, 3, and 7 days after release (DAR) in experiments conducted in March 2023 (fall; A, B, and C) and October 2023 (spring; D, E, and F) in Cordeirópolis, São Paulo State, Brazil. Each point represents the mean frequency of *D. citri*, with error bars indicating the standard error of the mean. The significance of Spearman rank order (rho) correlation values is also reported for the first experiment (panels A, B, and C), where a monotonic relationship was observed in the dataset.

**Table S1.** Total number of marked *Diaphorina citri* (Asian citrus psyllid) adults per plot of young sweet orange (*Citrus × sinensis* cv. Hamlin) trees grafted onto vigorous (‘Sunki Tropical’) and dwarfing (‘Flying Dragon’) rootstocks. Evaluations were conducted 1, 3, and 7 days after release (DAR) of marked psyllids in the experiments performed in March 2023 (fall) and October 2023 (spring) in Cordeirópolis, São Paulo State, Brazil. Data are presented as means ± standard error (SE).
**Table S2.** Total number of migrating marked *Diaphorina citri* (Asian citrus psyllid) adults moving between plots of young sweet orange (*Citrus × sinensis* cv. Hamlin) trees grafted onto vigorous (‘Sunki Tropical’) and dwarfing (‘Flying Dragon’) rootstocks. Evaluations were conducted 1, 3, and 7 days after release (DAR) of marked psyllids in experiments performed in March 2023 (fall) and October 2023 (spring) in Cordeirópolis, São Paulo State, Brazil. Data are presented as means ± standard error (SE).

## Data Availability

The data that support the findings of this study are available from the corresponding author upon reasonable request.
