# Supplementary material for: Rootstock impacts on citrus flush dynamics, vegetative growth, and Diaphorina citri infestation and dispersion: Implications for huanglongbing management
Source: Pest Manag Sci. 2026 May 21;82(9):8795–809. doi: 10.1002/ps.70929 (PMC13453183; doi:10.1002/ps.70929)
Supplement: Supplementary file 1 [file PS-82-8795-s001.docx]

**Fig. S1.** Map of the experimental area (panel A), young sweet orange trees (*Citrus × sinensis* cv. Hamlin) grafted onto dwarfing (‘Flying Dragon’) and vigorous (‘Sunki Tropical’) rootstocks, photographed 16 months after planting (panel B), and release platform positioned 4 m from the first row of planting, oriented towards the center of the plot, used for the release of marked *Diaphorina citri* during experiments conducted in fall (March 2023) and spring (October 2023) in Cordeirópolis, São Paulo State, Brazil. (Source: Google Earth)

**Fig. S2.** Map of the experimental area indicating the spatial distribution of sampled trees. For each plot, six trees were evaluated for *Diaphorina citri* capture using yellow stick cards (YSC) over time, and sixteen trees were assessed for vegetative growth, flush shoot dynamics and length, and fruit yield. Tree sampling was conducted in both vigorous and dwarfing rootstock treatments according to the designated layout within each plot. Circles indicate the trees assessed for specific measurements: blue, trees assessed for *D. citri* capture by YSC; red, trees assessed for vegetative growth and flush growth. All plots (*n* = 16) were evaluated for these parameters according to the rootstock, although not all individual trees are circled in the figure for clarity.

**Fig. S3.** Monthly average rainfall (mm) and air temperature (Max. Temp. = maximum temperature; Min. Temp. = minimum temperature; Mean Temp. = mean temperature) recorded at the experimental area located in Cordeirópolis, Southern São Paulo State, Brazil. Data span from September 2022 to April 2024.

**Fig. S4.** Daily rainfall and mean air temperature recorded during the release study of marked *Diaphorina citri* on young sweet orange trees (*Citrus × sinensis* cv. Hamlin) grafted onto vigorous (‘Sunki Tropical’) and dwarfing (‘Flying Dragon’) rootstocks. Data were collected during evaluations conducted 1, 3, and 7 days after release (DAR) in experiments held in the fall (March 2023) and spring (October 2023) in Cordeirópolis, São Paulo State, Brazil.

**Fig. S5.** Relationship between the frequency of marked *Diaphorina citri* and distance (m) from the release platform on young sweet orange (*Citrus × sinensis* cv. Hamlin) trees grafted onto vigorous (‘Sunki Tropical’) and dwarfing (‘Flying Dragon’) rootstocks at 1, 3, and 7 days after release (DAR) in experiments conducted in March 2023 (fall; panels A, B, and C) and October 2023 (spring; panels D, E, and F) in Cordeirópolis, São Paulo State, Brazil. Each point represents the mean frequency of *D. citri*, with error bars indicating the standard error of the mean. The significance of Spearman rank order (rho) correlation values is also reported for the first experiment (panels A, B, and C), where a monotonic relationship was observed in the dataset.
