# Supplementary material for: Rootstock impacts on citrus flush dynamics, vegetative growth, and Diaphorina citri infestation and dispersion: Implications for huanglongbing management
Source: Pest Manag Sci. 2026 May 21;82(9):8795–809. doi: 10.1002/ps.70929 (PMC13453183; doi:10.1002/ps.70929)
Supplement: Supplementary file 2 [file PS-82-8795-s002.docx]

**Table S1.** Total number of marked *Diaphorina citri* (Asian citrus psyllid) adults per plot of young sweet orange (*Citrus × sinensis* cv. Hamlin) trees grafted onto vigorous (‘Sunki Tropical’) and dwarfing (‘Flying Dragon’) rootstocks. Evaluations were conducted 1, 3, and 7 days after release (DAR) of marked psyllids in the experiments performed in March 2023 (fall) and October 2023 (spring) in Cordeirópolis, São Paulo State, Brazil. Data are presented as means ± standard error (SE).

| Days after release (DAR) | Vigorous | Dwarfing | *P*-value |
| --- | --- | --- | --- |
| *March 2023 (Fall)* |  |  |  |
| 1 DAR | 19.5 ± 4.9 b^†^ | 37.3 ± 7.4 a | 0.043 |
| 3 DAR | 43.8 ± 6.8 a | 45.6 ± 6.3 a | 0.686 |
| 7 DAR | 27.0 ± 3.0 a | 32.8 ± 4.1 a | 0.251 |
| *October 2023 (Spring)* |  |  |  |
| 1 DAR | 21.5 ± 5.5 a | 4.1 ± 1.3 b | 0.013 |
| 3 DAR | 24.3 ± 6.6 a | 5.0 ± 1.9 b | 0.015 |
| 7 DAR | 15.8 ± 3.9 a | 2.5 ± 0.6 b | 0.012 |

^†^Means followed by the same letter in the row are not significantly different according to ANOVA (F-test).

**Table S2.** Total number of migrating marked *Diaphorina citri* (Asian citrus psyllid) adults moving between plots of young sweet orange (*Citrus × sinensis* cv. Hamlin) trees grafted onto vigorous (‘Sunki Tropical’) and dwarfing (‘Flying Dragon’) rootstocks. Evaluations were conducted 1, 3, and 7 days after release (DAR) of marked psyllids in experiments performed in March 2023 (fall) and October 2023 (spring) in Cordeirópolis, São Paulo State, Brazil. Data are presented as means ± standard error (SE).

| Days after release (DAR) | Vigorous | Dwarfing | *P*-value |
| --- | --- | --- | --- |
| *March 2023 (Fall)* |  |  |  |
| 1 DAR | 0.8 ± 0.3 | 1.8 ± 0.7 | 0.154 |
| 3 DAR | 2.5 ± 0.5 a | 1.8 ± 0.3 b | 0.048 |
| 7 DAR | 2.4 ± 0.8 a | 1.0 ± 0.4 b | 0.037 |
| *October 2023 (Spring)* |  |  |  |
| 1 DAR | 3.1 ± 1.2 | 2.5 ± 0.9 | 0.660 |
| 3 DAR | 0.7 ± 0.3 | 0.4 ± 0.2 | 0.289 |
| 7 DAR | 0.9 ± 0.5 | 0.5 ± 0.3 | 0.095 |
